# The annual feasibility and affordability of a healthy diet for families with children in Israel by income quintile and geographic area of residency

**DOI:** 10.1186/s13584-025-00675-7

**Published:** 2025-03-27

**Authors:** Naama Dgania-Yaroslaviz, Moran Blaychfeld Magnazi, Vered Kaufman-Shriqui

**Affiliations:** 1 Faculty of Health Sciences, Department of Nutrition Sciences, Ariel University, Kiryat Hamada 3, Ariel, Israel; 2 The Center for Urban Health Solutions (C-UHS), St., Michael’S Hospital, Toronto, Canada; 3 Nutrition Division, Ministry of Health, Jerusalem, Israel; 4 School of Public Health, Haifa University, Haifa, Israel

**Keywords:** Child nutrition, Food costs, Health disparities, Nutrition policy, Socioeconomic factors

## Abstract

**Background:**

Dietary guidelines for families with children are designed to meet the Dietary Recommended Intake. However, the cost of a healthy diet and the extent to which families can afford it in Israel is unclear.

**Methods:**

The age distribution and the number of children per household by income quintile and geographic area in Israel in 2018 were obtained from the Central Bureau of Statistics. Food cost information was purchased from the commercial company Stornext. The cost of the recommended food items in the healthy diet for adults and children (by age group) was calculated using standard food portions and meal frequency and expressed as a percentage of the households net income. The proportion of households for which food expenditures exceeded 15% of the net income was calculated, followed by changes in food prices during 2018.

**Results:**

The average daily dietary cost per person was 35.5 ± 7.7 New Israeli shekels (equivalent to $9.7 ± $2.11). For households with children, the median monthly cost of the recommended diet, as a percentage of net household income was 20%. There was an inverse association with socioeconomic status, as the median monthly food expenses for the first (lowest) quintile were 55% of the household's net income and only 9.3% of the 5th (highest) income quintile. By geographic residential area, the median percentage of the net income from monthly dietary costs was 23%. The highest costs were in Judea, Samaria, and Jerusalem. Lunch made up 47% of food expenditures, if theoretically omitted, diet expenditures for households with children would decrease by an average of 15%. The food group that composed the highest component of the food budget was the vegetable group, with an average cost of 29% monthly, followed by the meat and meat substitutes group (19%).

**Conclusion:**

This theoretical calculation shows that two-thirds of the households with children in Israel could not purchase the recommended diet in 2018, with significant disparities according to socioeconomic status. Policymakers should consider steps to decrease health inequality in food affordability, targeting the three middle-lower income quintiles. Our findings suggest the need to expand the provision of school lunches. Further research is required to examine how changes in household food costs influence consumers’ food choices and the potential health implications of the high expenses identified in this study.

## Background

Access to nutritious food is essential for health across all life stages. Economic and accessibility challenges significantly impact diet quality, especially among low-socioeconomic status (LSES) families, perpetuating nutritional disparity [1, 2]. Food choices are shaped by affordability, availability, and accessibility [3]. LSES families face difficulty affording healthier options, leading to lower diet quality and adverse health impacts, including malnutrition and non-communicable diseases [1, 2, 4–6].

Internationally, rising food prices, especially fruits and vegetables, have increasingly restricted access to nutritious diets for LSES families. This has been documented in Israel as well [7–12]. From 2005 to 2020, the Consumer Price Index for these items rose substantially, creating economic barriers for those striving to purchase these essentials [13]. In contrast, higher-income households can typically afford these healthier, costlier foods [7].

Food insecurity (FI), defined as limited access to nutritionally adequate foods [6], remains a critical issue in Israel. In 2021, 16.1% of Israeli families, including a fifth of children, experienced FI [14]. The Taub Center’s 2016 Healthy Food Basket (HFB) report highlighted a gap, showing that while higher-income households exceeded the HFB’s estimated cost, lower-income households (1–3 quintiles) could not meet it, indicating a price barrier to a balanced diet [15]. However, the report lacked geographic data and relied on commercial data from major food chains, overlooking price variations and access challenges for those without convenient access to such stores.

Accessibility barriers further exacerbate food security issues, as limited access to supermarkets and affordable sources of nutritious food makes maintaining a balanced diet incredibly challenging for lower-income families. While Israel has not formally documented “food deserts,” approximately 30% of households lack private transportation, restricting access to more prominent, affordable grocery outlets and highlighting how economic and logistical constraints shape dietary choices [16–18].

Household food expenditure in Israel reflects these disparities. In 2018, households allocated 14.9% of their income to food, 12.2% for grains, 7.5% for fruits, 10.7% for vegetables, and 19.9% for meat, poultry, and fish [16]. However, this spending pattern varies widely across socioeconomic levels, affecting diet quality. While policies like the "hot meal program" support children in FI situations by providing one hot meal daily at schools [19, 20], they do not fully address the broader issue of accessibility to nutritious foods for all families.

Using the most updated available data from the Central Bureau of Statistics and purchasing data, this study aims to update and expand previous knowledge and estimate the cost of a healthy diet in Israel compared to actual household food expenditure across different socioeconomic groups, income quintiles, and geographic areas. By examining the economic and accessibility factors influencing diet quality, the study may suggest insights that could inform policies to enhance access to nutritious foods and promote nutritional equity.

## Methods

### Data sources and study design

**Fig. 1:**
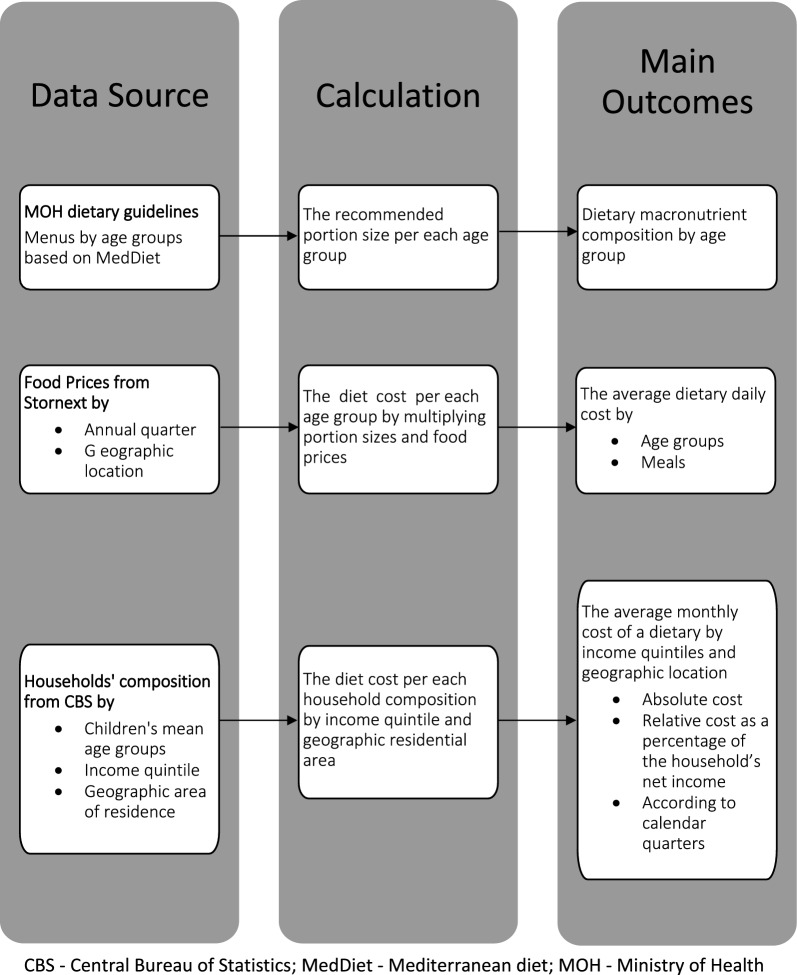
Study design

The present study is an ecological correlational study relying on existing data. Our primary data were obtained from administrative sources, including household composition, recommended diets, and food prices. Figure 1 presents the study design. Dietary guidelines for children's daily nutritional needs, including food portions and recommended frequency of food items, are based on the Israeli Ministry of Health (MOH) guidelines for healthy eating [21–23]. Using the food products included in the Israeli HFB, we calculated the cost of a healthy diet by age group. Household composition and income quintiles were obtained from the Central Bureau of Statistics (CBS) [24].

#### Israeli dietary guidelines

We used dietary guidelines from published reports and guidelines of the Israeli MOH for children and adults by age group. Briefly, the Israeli nutritional guidelines [25] are based on the Mediterranean diet, which is associated with lower morbidity and mortality at all ages [26–28]. The Mediterranean diet is generally characterized by a high intake of plant foods (e.g., vegetables, fruits, legumes, nuts, preferably whole-grain cereals), use of olive oil as the main dietary fat, a moderate intake of dairy products, a low or moderate intake of meat, poultry and fish, and a moderate consumption of wine (irrelevant for children). In addition, the Mediterranean diet is characterized by using fresh and local ingredients, home cooking, lower amounts of processed foods, and, thus, higher sustainability [29, 30]. For this research, we used menus prepared by the MOH for educational frameworks supplying meals for children based on the Mediterranean diet. The diets included food (items of food groups), variability, portion sizes, and frequency by food groups (times per week). The amounts of food items (measured in grams) were defined in the diets according to the following age groups: 1–3 [21], 4–5 [21], 6–8 [22], 9–13 [22], 14–18 [22], and 18–64 [23] years. The diets were composed of specific age groups.

#### Household composition

Using the latest Israeli census data (2018) of 2,549,070 households, the CBS analyzed the data according to our research needs and defined the average number of children per income quintile and geographic area by aggregating the numbers and percentages by children's age categories. (Appendix 1). For our analysis, we requested a special report on children's ages according to the ages of the recommended diets from CBS. Children aged 1–18 years were included in the analysis. Children younger than one year were excluded from the calculations since using a milk substitute (i.e., baby formula) is not the nutritional recommendation of the World Health Organization (WHO) and because there are no standards on how to price a breastfeeding mother's diet [21].

#### Food prices

We obtained food price data by geographic area and annual quarters per year from the commercial company Storenext [31]. The barcode contained the prices of products in the recommended HFB (64 items). For products not included in the basket and with no specific barcode, prices were matched according to the most frequently sold food item in this category by region and for each quarter of 2018. The 64 items from the HFB constituted 54% of the overall food items included in this analysis, and the remaining 46% of the items are the cheapest in their category which could be found in Stornext data.

Total food costs were then summed by family composition and expressed as a percentage of the net household income. The results are presented by household income quintile and geographic area. Databases and prices are updated to the latest available CBS survey of families with children from 2018 [16].

### Calculating the dietary cost for households with children

#### Composition of the diet

The MOH and MOEI included foods in the HFB according to consumption data, as reflected in the Food Frequency Questionnaire (FFQ) of the 2014–2016 health and nutritional status survey. In each category, healthier alternatives were chosen [32]. We applied the following additional considerations to reduce dietary costs:

1. For products included in the HFB, the price was calculated based on the average price according to the barcode of the product selected for monitoring in the HFB 2022
2. For products not included in the HFB, the most common, best-selling food brand was selected (i.e., "Osem" was selected as the most-sold brand of pasta).
3. Expensive meats were not included in the food group of "animal protein and legumes." Instead, cheaper products such as frozen whole chicken and frozen chicken breast were included.
4. A recipe was created for complex foods, such as pizza orcake, and the ratio of the main ingredients was calculated. Portion sizes were exacted according to those in the designated diets.
5. In the vegetable group, we used the average price for the specific common vegetables included in the HFB:
6. Fresh vegetables—scallions, lettuce, carrots, cucumbers, button mushrooms, white cabbage, tomatoes, red peppers, radishes, kohlrabi.
7. The cooked vegetables used were squash, cilantro, carrots, parsley, zucchini, and celery leaves.

#### Calculation of the cost of diets

Based on the recommended diets for adults and children aged one to 18, we multiplied serving sizes by the frequency of food items appropriate to the age group. Because the recommended daily intake in each group is composed of several foods, each serving represented a relative average of several foods in the same group. We calculated the total weight and price per week for each food item.

#### Quality control

Prior to calculating the costs, we compared the diets for each age group based on the MOH according to the DRIs. The nutritional requirements for energy were determined according to the Food and Agriculture Organization (FAO) guidelines [33]. The requirements for protein, carbohydrates, and micronutrients were calculated according to the DRIs [34]. Appendix 1 shows the composition of the diets per age group.

#### The food cost for households by income quintiles

To calculate the monthly household cost for each income quintile, we first calculated the monthly cost of each meal and each age group per person. We then expressed the diet cost per household as a percentage of net income per quintile. We multiplied the total number of people in each income quintile and age group and calculated the prices of the meals per day.

#### Household dietary cost by geographic area of residence

The subdivisions of geographic areas according to CBS data were as follows: Jerusalem, North, Haifa, Central, Tel Aviv, South, Judea, and Samaria. The subdivisions of geographic areas according to the Storenext database were as follows: Jerusalem, North except Haifa; Haifa, South Central, North Central, Tel Aviv, and South. Judea and Samaria are included in Jerusalem, North Central, and South. Food prices were adapted to the CBS geographic area by checking the matching of the places.

To calculate the monthly food costs for a household divided into seven geographic areas, we multiplied the cost of the meal per person for each age group by the number of people per geographic area. We expressed the dietary costs as a percentage of net income according to the 2018 CBS data.

#### The proportion of the dietary costs of the household's income

The cost was divided by the household's net income to examine households with children's theoretical ability to finance monthly dietary costs. Patient demographic groups and income levels were also included. Daily costs were calculated by average age and national average price and are expressed as a percentage of each food group. The average price of a daily healthy diet by age group was calculated, as was the average cost of a healthy diet per 1,000 cal by age group.

### Dietary costs by price trends during 2018

The cost of a healthy diet per household and quintile according to food price trends in 2018 is described by annual quarters. The first quarter includes January, February, and March. The second quarter includes April, May, and June; the third includes July, August, and September; and the fourth includes October, November, and December. The seasonality of fruits and vegetables was considered.

### Statistical analysis

The statistical analysis was performed using IBM SPSS statistics 27. We performed the Kolmogorov‒Smirnov test to check the normality of the distribution of food prices. The mean food costs are presented as the mean ± SD or median values. Proportions are presented as rates and percentages. We used Spearman's rank correlation coefficient to estimate the correlation between the income quintile and the percentages of the dietary costs of the income. Additionally, a t-test for independent samples was performed to determine if a statistically significant difference (P < 0.05) existed between the cost of a vegetarian diet (VegDiet) and a regular diet. The correlation could theoretically vary between 0 and 1.0. A correlation of 0 indicates a different price, whereas a correlation of 1.0 indicates no difference in price. Correlation values reflect a low match below 0.40, a fair to moderate game from 0.4 to 0.75, and a high match greater than 0.75. A P value of 0.05 indicated statistical significance.

## Results

### The daily dietary cost by age group

The average daily dietary cost per person was 35.5 ± 7.7 NIS, with variations across meals, age groups, and areas of residency. As shown in Table 1, the dietary costs increased with age. The cost of breakfast for individuals aged 9–13 is lower than for younger people because there is no recommendation for yogurt compared to age groups 1–8.

**Table 1:** Average price ± SD by meal and age group (NIS)

Age group (years)	1–3	4–5	6–8	9–13	14–18	19–64	Mean (NIS)	SD
Breakfast	7.03	7.01	7.02	6.28	7.00	10.82	7.53	1.50
Lunch	8.55	12.57	14.12	18.98	20.44	22.94	16.27	4.95
Dinner	3.99	5.44	4.55	4.76	5.61	9.31	5.61	1.74
Intermediate* (Two meals)	3.38	6.61	6.78	7.60	7.60	4.67	6.11	1.56
The total cost of meals per day	22.94	31.63	32.47	37.62	40.64	47.73	35.51	7.77

* Intermediate meals are usually eaten at 10 am and 4 pm in a typical Israeli schedule

### Variation in the average diet cost between different age groups

**Fig. 2:**
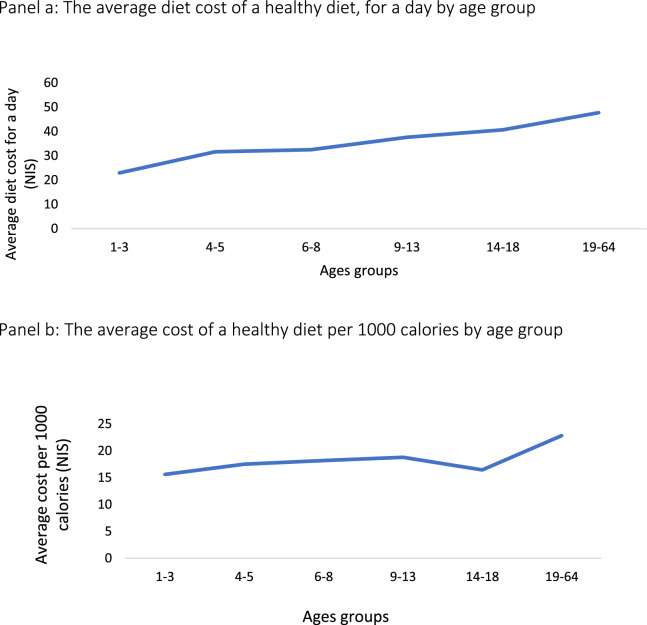
Variation in the average diet cost between different age groups

We found a direct association between an increase in age group and an increase in the average price of a healthy daily diet (ρ = 1, p < 0.01), but no significant difference was found after further adjustment for 1000 cal (except for the age group of children aged 14–18 years) (ρ = 0.657, p = 0.156) (Fig. 2). The cost increased by 1.7 from 23 NIS for children aged 1–3 years to 41 NIS for children aged 14–18.

### Actual expenditure on food per family vs. the recommended expenditure on a healthy diet by income quintile, 2018

**Fig. 3:**
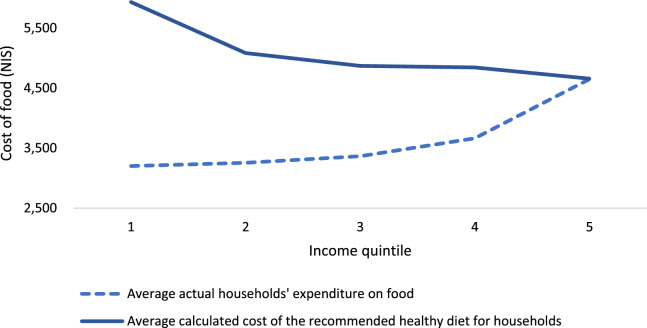
Actual expenditure on food per family vs. the cost of the recommended healthy diet by income quintile, 2018

Figure 3 shows the difference in shekels per household between the required expenditure on food, according to our calculations, and the actual expenditure on food, as we received from the Central Bureau of Statistics, for all income quintiles. The largest gap exists in the first quintile (2700 NIS), and the increase in income quintiles reduces the gap.

### The cost of a healthy diet for households with children by income quintiles

**Fig. 4:**
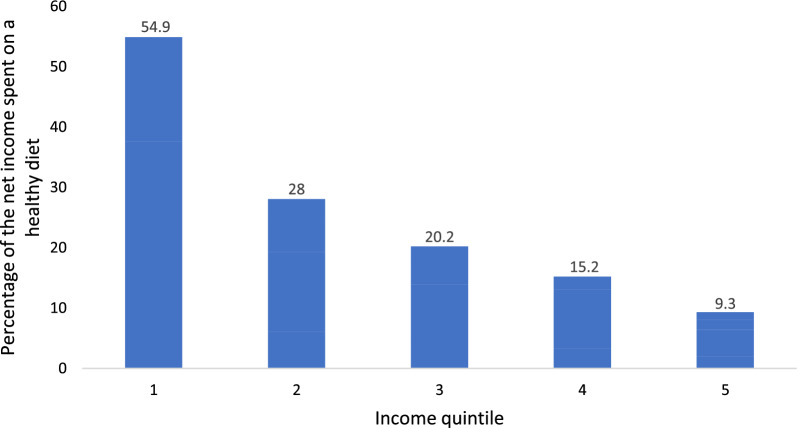
The monthly cost of a healthy diet as a percentage of the household net income

The median monthly dietary expense for households with children as a percentage of the net income was 20% (approximately 5,000 NIS per month). An inverse significant association between SES and BMI was found (ρ = −1, p < 0.001). As shown in Fig. 4, the costs of the recommended healthy diet from net income decrease as income quintiles increase: 55% of the first (lowest) income quintile and 9.3% of the 5th (highest) income quintile.

### Cost of a healthy diet for households with children by geographic residential area

The median monthly food cost for a household with children was 23% of its net income, with differences across meals and geographic residency areas. No statistically significant relationship was found between geographic residential location and the costs of the healthy menu for the household out of the net income (χ = 42, p = 0.227). The highest costs across all locations were attributed to lunch. The cost of lunch was 47% of the overall food expenses. The highest costs were found in Judea, Samaria, and Jerusalem (5780 NIS and 5700 NIS, respectively), and the lowest were found in Haifa and the North (4900 NIS and 4860 NIS, respectively). The costs in Tel Aviv, the Center, and the South ranged from 4960 to 5350 NIS.

### Tracking Trends in Food Basket Prices During 2018

**Fig. 5:**
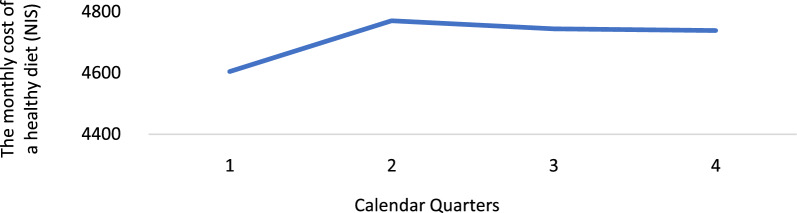
Description of average healthy diet costs by calendar quarters, 2018

As shown in Fig. 5, the time trend evaluation of the cost of a healthy diet revealed an increase from the first quarter to the second (ρ = 1, p < 0.01), followed by a plateau until the fourth quarter (ρ = 0.651, p = 0.349).

### Price proportions according to food groups

**Fig. 6:**
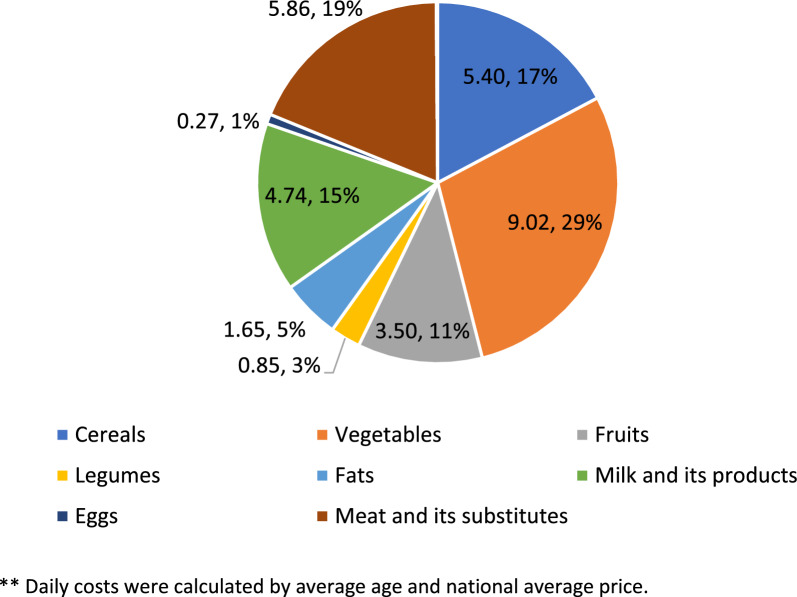
Percentage of daily cost by food group from the total price of an average diet** **Daily costs were calculated by average age and national average price

As presented in Fig. 6, costs varied by food group. The mean cost of the vegetable group was the highest component of the food budget, with an average daily price of 29% (9.02 NIS per day), followed by the meat and meat substitute group (19%). The group that was priced less was eggs and legumes (< 1%).

### The cost of a vegetarian diet

An additional calculation examined the cost of a vegetarian diet (in accordance with the recommendation of the MOH guidelines for a vegetarian diet) [21, 22]. We found a high correlation of 0.995 (P < 0.001) and no differences compared to a regular diet. The mean was −0.21 less expensive for the regular diet [95% confidence interval (95% CI) –13.25, 13.43], P = 0.98].

### The average percentage monthly food cost by meal for households, 2018

**Fig. 7:**
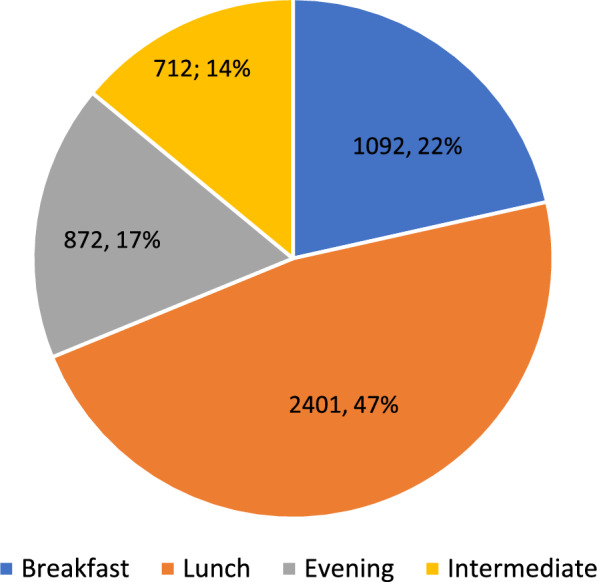
The average monthly cost by meal for households (NIS as a percentage of the income)

Figure 7 presents the average cost of meals. The ratio between the average cost of breakfast, lunch, dinner, and two intermediate meals was 1.5:3.4:1.2:1, respectively, with lunch being the most expensive.

### The cost of a healthy diet for households with children by income quintiles, theoretically after omitting the cost of lunch for children during the five days of the week

**Fig. 8:**
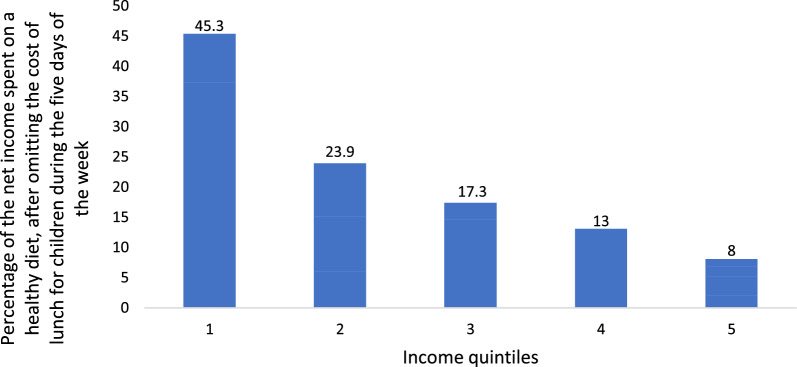
Healthy Diet Monthly Cost as a Percentage of Household Income, Excluding Children’s Weekday Lunch Expenses

Since 2018, a few changes have occurred in terms of eligible SFP children. In addition, if the SFP is expanded as recommended by Israeli experts to include all school-age children, they should receive lunches at educational institutions [35], and after theoretically omitting the cost of lunch for children during the five days of the week (Fig. 8), the median monthly dietary expense for households with children as a percentage of the net income should be 17% (approximately 4,100 NIS per month). Compared to the cost of a healthy diet that includes lunch for children during the seven days of the week (Fig. 4), there would be, on average, a 15% decrease in diet expenditures for households with children.

### Food price index

In Israel, from January 2018 to December 2022, the food price index, including vegetables and fruits, increased by 11.4% [36]. The average cost of a recommended healthy diet per household in 2018 was 5000 (± 449) NIS. It can be estimated that in 2022, the recommended healthy diet per household cost was 5570 (± 500) NIS. Since we know that the level of net income for households has also risen accordingly, we assume that the proportion of expenditure on food out of income has remained the same throughout the years 2018–2023 for all income quintiles [36].

## Discussion

This study quantified the cost of following governmental dietary guidelines for Israeli households with children, revealing an average monthly expense of 5000 (± 449) NIS in 2018. On average, this represented about 25% of household income, with stark disparities across income levels: 54.9% for the lowest income quintile and 9.3% for the highest (Appendix 2.1 CBS data). Unlike the Taub Center’s 2016 Healthy Food Basket (HFB) report, which used general dietary recommendations, our study utilized age-specific menus for a more precise cost assessment. It included regional price variations that the Taub report did not address [15]. These regional differences were evident, with higher costs in Jerusalem, Judea, and Samaria compared to lower costs in Haifa and the North [16]. Families in regions with larger household sizes, like Jerusalem, faced even higher costs despite lower average expenses in other areas, emphasizing the compounded impact of both family size and location on affordability (Appendix 2.2 CBS data). Our study also accounts for seasonal trends in food prices, showing quarterly cost fluctuations throughout 2018, an insight missing in prior reports yet essential for policy planning responsive to annual and seasonal changes [36].

Our findings reveal significant income-based disparities, with low-income families spending 54.9% of their income on a healthy diet, compared to 9.3% for high-income households [16]. We also identified that Israeli families with children allocate a considerable portion of their income to food. In 2018, the daily cost of a healthy diet per individual was approximately 11.3 USD, almost three times higher than the 4.3 USD average in high-income countries [37]. The cost ratio of a healthy diet to actual food expenditure was 1.38 in Israel, far exceeding the 0.77 seen in other high-income nations, underscoring the affordability challenge [38]. Additionally, lunch was the most expensive meal, making up 47% of the daily food cost, which suggests targeted policies to expand school meal programs [19, 39].

School meal programs play a crucial role in promoting healthy eating habits among children, as shown in the U.S. and U.K., where participants in school feeding programs have been found to consume less saturated fat and sugar, with healthier weight outcomes than non-participants [40–42]. In Israel, where lunch is often the most costly meal, the School Feeding Program (SFP) was established in 2005 to provide daily meals to eligible students, although access remains limited. Expanding this program could further support children’s health, educational attainment, and social equity, particularly for families in the middle-income deciles [19, 39, 43]. Additionally, implementing mechanisms to ensure meal access during school closures could prevent food insecurity during crises [20]. Expanded access to school meals could reduce household food costs significantly, with government-funded lunches previously decreasing food expenses by approximately 900 NIS per month, or 15%, for families with children in 2018.

Addressing affordability and accessibility is essential to tackle food insecurity. Research has shown that low-income households are more likely to buy unhealthy foods due to their lower cost and greater availability than healthier options [44, 45]. Policies to make nutritious foods more affordable include subsidies, price adjustments, and government-regulated healthy food baskets [46]. Another policy tool is the development of a healthy food basket, which varies across countries based on ethnic/local flavors, typical consumption, and health considerations. For instance, Australia's Illawarra Healthy Food Basket (IHFB) includes 57 items [47], while Canada's 2019 food basket includes 61 nutritious foods [48]. In 2020, Israel's Ministry of Health (MOH) and Ministry of Economic and Industry (MOEI) launched a Healthy Food Basket (HFB) of 64 nutritionally recommended products based on the Mediterranean diet, providing a framework for nutritious, affordable diets [49].

This study’s strengths include a rigorous calculation of healthy diet cost based on MOH-recommended diets by age group, exact food portions, no food waste, no special meals or take-out, and no hospitality expenditures, making our estimates conservative. We did not include drinking costs, assuming tap water consumption [50]. However, the study also has limitations, including reliance on 2018 price data, with food prices increasing by 11.4% since then [36], leading to underestimated current costs and a theoretical approach that excludes personal and cultural factors influencing food choices. Geographic cost calculations were limited in Judea and Samaria due to insufficient food outlets, grouping them with nearby regions.

## Conclusions

This study highlights substantial gaps in affordability for healthy diets across income and geographic areas. These findings provide a basis for policymakers to track food prices regionally and implement targeted policies, such as expanding school meal programs and adjusting nutritional guidelines that promote equitable access to nutritious foods for all Israeli households. Further research is needed to monitor food costs over time and explore the broader health impacts of these affordability challenges, specifically on families with children.

## Data Availability

The datasets used and/or analyzed during the current study are available from the corresponding author upon reasonable request.

## Apppendix

See Tables 2, 3 and 4

**Table 2:** Distribution of macronutrierrts by age group as percentage of the diet

	Protein (Range of valid values*)	Total lipid (fat) (Range of valid values*)	Carbohydrates (Range of valid values *)
Ages 1–3	18%	30%	52%
	(5%–20%)	(30%–40%)	(45%-65%)
Ages 4–5	19%	25%	56%
	(10%-30%)	(25%-35%)	(45%-65%)
Ages 6–8	20%	30%	50%
	(10%–30%)	(25%-35%)	(45%-65%)
Ages 9–13	20%	30%	50%
	(10%-30%)	(25%-35%)	(45%-65%)
Ages 14-1S	20%	29%	51%
	(10%-30%)	(25%–35%)	(45%-65%)
Ages 18–64	16%	25%	49%
	[10%–35%]	(20%-35%)	(45%-65%)

*Range of valid values is by DRI [42]

**Table 3:** Reference family by income quintile, 2018*

	Quintile					Mean	SD
	1	2	3	4	5		
Number of children in the family	3.2	2.3	2.1	2.0	1.8	2.4	0.5
Average Net Wage (NIS)	10,804	18,104	24,068	31,836	49,985	3,519	3,429.40
	13.741	17,362	19,896	24,154	31,377	9,751	6,071.5
Average monthly consumption expenditure (NIS)							
Average expenditure on food {NIS)	3,202	3,254	3,365	3,664	4,649	3,499	535.5
% Expendrture on food	29.64	17.98	13.98	11.51	9.30	14.88	7.2

*Data from the CBS (2018) the food expend itu re including fru its and vegeta bles

**Table 4:** Reference family by geographic residential of-area, 2018*

	Geographical district							Mean	S. D
	Jerusalem	North	Haifa	Central District (without Tel Aviv)	Tel Aviv	South	Judea and Samaria		
Number of children In the family	3.0	2.2	2.2	2.2	2.1	2.7	3.2	2.4	0.45
Average Net Wage (NIS)	19,295	8,790	22,421	9,511	6,306	1,074	1,601	3,519	3877.35
Average monthly consurnption expenditure (NIS)	17,261	17,693	19,478	22,128	22,082	18,565	18,281	19,751	2002.34
Average expenditure on food (NIS)	3,367	3,721	3,683	3,387	3,398	3,582	3,238	3,499	181.00
% Expenditure on food	17.45	19.80	16.48	11.48	12.92	17.00	14.99	14.88	2.83

*Data from the CBS (2018): the food expenditure including fjuits and vegetable

## Abbreviations

CBS: Central Bureau of Statistics
CPI: Consumer Food Price Index
DRI: Dietary Reference Intake
FAO: Food and Agriculture Organization
FNS: Food and Nutrition Service
FFQ: Food Frequency Questionnaire
GDP: Gross Domestic Product
HFB: Healthy Food Basket
HEISB: Healthy Eating Indicator Shopping Basket
IHFB: Illawarra Healthy Food Basket
MedDiet: Mediterranean Diet
MOH: Ministry of Health
MOEI: Ministry of Economy and Industry
NSLP: National School Lunch Program
NIS: New Israeli Shekel
NGOs: Nonprofit Organizations
SES: Socio-Economic Status
SDG: Sustainable Development Goals
SFP: School Feeding Program
USDA: United States Department of Agriculture
USD: US Dollar
VegDiet: Vegetarian Diet

## References

[1] World Health Organization. Malnutrition. 2021; Available at: https://www.who.int/health-topics/malnutrition#tab=tab_1. Accessed 01.12.2021.

[2] WinStar Communications paid $17 million for 47 additional 38 GHz licenses in major cities. Communications Daily. 1997 Feb;17(22):5.

[3] Ruel M. Food Security and Nutrition: Linkages and Complementarities. The Road to Good Nutrition Basel, Switzerland: S. Karger AG; 2013. p. 24–38.

[4] World Health Organization. Obesity and overweight. 2021; Available at: https://www.who.int/news-room/fact-sheets/detail/obesity-and-overweight Accessed 9.8.2024

[5] Baer-Nawrocka A, Sadowski A. Food security and food self-sufficiency around the world: A typology of countries. PLoS ONE. 2019;14(3): e0213448. 30845273 10.1371/journal.pone.0213448 PMC6407907

[6] Quality Indicators. (n.d.) National Program for Quality Indicators in Community Healthcare in Israel: Report. 2017. Available at: https://48fc89f4-e14d-48de-bdc0-ec96de79873e.filesusr.com/ugd/76a237_839988734c8a44d4822384e11afa6c0a.pdf Accessed 9.8.2024

[7] Downs SM, Fox EL, Zivkovic A, Mavros T, Sabbahi M, Merchant EV, et al. Drivers of food choice among women living in informal settlements in Nairobi. Kenya Appetite. 2022;168: 105748. 34637773 10.1016/j.appet.2021.105748

[8] Darmon N, Lacroix A, Muller L, Ruffieux B. Food price policies improve diet quality while increasing socioeconomic inequalities in nutrition. IJBNPA. 2014;11(1):66. 24886414 10.1186/1479-5868-11-66 PMC4045909

[9] Lee A, Mhurchu CN, Sacks G, Swinburn B, Snowdon W, Vandevijvere S, et al. Monitoring the price and affordability of foods and diets globally. OBES REV. 2013;14(S1):82–95. 24074213 10.1111/obr.12078

[10] Monsivais P, Aggarwal A, Drewnowski A. Are socio-economic disparities in diet quality explained by diet cost? J Epidemiol Community Health. 2012;66(6):530–5. 10.1136/jech.2010.122333. 21148819 10.1136/jech.2010.122333 PMC3951975

[11] Swinburn BA, Kraak VI, Allender S, et al. The Global Syndemic of Obesity, Undernutrition, and Climate Change. The Lancet. 2019. 10.1016/S0140-6736(19)30384-8]. 30700377 10.1016/S0140-6736(18)32822-8

[12] Hirvonen K, Bai Y, Headey D, Masters WA. 2020 Affordability of the EAT-Lancet reference diet: a global analysis [published correction appears in Lancet Glob Health. 10.1016/S2214-109X(20)30472-1]. 10.1016/S2214-109X(19)30447-4 PMC7024996 31708415

[13] State Comptroller of Israel. Annual Audit Report 71c | Systemic issues. The treatment of monopoly and centralization in the food industry. 2021. Available at: https://www.mevaker.gov.il/sites/DigitalLibrary/Documents/2021/71C/2021-71c-101-Mazon.pdf 2021. Accessed 9.8.2024

[14] Andeblad M and laave C. Food security survey 2021.

[15] The course of the survey and the main findings. 2021. Available at: https://www.btl.gov.il/Publications/research/Documents/bitconTzonti%202021.pdf 2021. Accessed 9.8.2024

[16] Azarieva J, Orion B, Goldsmith R, Chernichovsky D. A Healthy Food Basket in Israel. Jerusalem: Taub Center for Social Policy Studies in Israel 2016.

[17] Alfandari Y, Dopaz L, Yosef C, Cohen- Shabtai H, Dahlika G, Mizrahi O, et al. Household income and expenditure data from the 2018 household expenditure survey general summary. 2020 October:79–83. Available at: https://www.cbs.gov.il/he/publications/DocLib/2020/1802_households_2018/e_print.pdf Accessed 9.8.2024.

[18] Economic Research Service, U. S. Department of Agriculture. Food Access Research Atlas - Documentation. 2021; Available at: https://www.ers.usda.gov/data-products/food-access-research-atlas/documentation/. Accessed 9.8.2024

[19] Huyghe E, Verstraeten J, Geuens M, Van Kerckhove A. Clicks as a Healthy Alternative to Bricks: How Online Grocery Shopping Reduces Vice Purchases. J Mark Res. 2017;54(1):61–74.

[20] Vurgen Y. Implementation of the daily meal law for students - a snapshot. Knesset. 2009 Nov. Available at: https://fs.knesset.gov.il/globaldocs/MMM/a35b6b58-e9f7-e411-80c8-00155d010977/2_a35b6b58-e9f7-e411-80c8-00155d010977_11_9008.pdf Accessed 9.8.24.

[21] Ministry of Health, Nutrition Division, Israel. Eat and grow: instructions for daycare centers from 3 months to 3 years of age and for multi-purpose daycare centers up to 5 years of age. 2020; Available at: https://www.health.gov.il/PublicationsFiles/eatandgrow.pdf Accessed 31.7.24

[22] Ministry of Health, Nutrition Division, Israel. Nutrition and food procedure in summer camps and children and youth camps. 2016; Available at: https://www.health.gov.il/hozer/BZ08_2016.pdf Accessed 31.7.24.

[23] Ministry of Health, Nutrition Division, Israel. A guide to sensible nutrition and a healthy lifestyle. Available at: https://www.gov.il/BlobFolder/reports/nutrition-mental-health-care/he/files_publications_units_Nutrition_nutrition_mental_health_care.pdf Accessed 31.7.24

[24] Central Bureau of Statistics. Household income and expenditure. Data from the household expenditure survey, 2018. Available at: https://www.cbs.gov.il/he/publications/DocLib/2020/1802_households_2018/h_print.pdf Accessed 9.8.24

[25] Ministry of Health. Israel's New National Nutrition Recommendations – the New Nutritional Rainbow Ministry of Health. 2020 Available at: https://www.gov.il/en/pages/dietary-guidelines Accessed 9.8.2024.

[26] Trichopoulou A, Costacou T, Bamia C, Trichopoulos D. Adherence to a mediterranean diet and survival in a greek population. NEJM. 2003;348(26):2599–608. 12826634 10.1056/NEJMoa025039

[27] Guasch-Ferré M, Willett WC. The mediterranean diet and health: a comprehensive overview. JIM. 2021;290(3):549–66. 10.1111/joim.13333 34423871

[28] López-Gil JF, García-Hermoso A, Martínez-González MÁ, Rodríguez-Artalejo F. Mediterranean diet and cardiometabolic biomarkers in children and adolescents. JAMA Netw Open. 2024;7(7): e2421976. 38995643 10.1001/jamanetworkopen.2024.21976 PMC11245727

[29] Álvarez-Álvarez L, Vitelli-Storelli F, Rubín-García M, Martín-Sánchez V, García Fernández C, Carvalho C, et al. Environmental impact of the diet of young Portuguese and its relationship with adherence to the Mediterranean Diet. Eur. J. Clin. Nutr. 2024 May. 10.1007/s00394-024-03396-w PMC11377495 38763928

[30] Dernini S, Berry EM, Serra-Majem L, La Vecchia C, Capone R, Medina FX, et al. Med Diet 4.0: the Mediterranean diet with four sustainable benefits. Public health nutrition. 2017 May;20(7):1322–1330. 10.1017/S1368980016003177 PMC10261651 28003037

[31] Storenext - To give business communities knowledge that serves as a solid basis for decision making. Available at: https://www.storenext.co.il/. Accessed 9.8.2024

[32] The Ministry of Economy and Industry, the Ministry of Health, and the Israel Consumer Council Launch a New Tool for Monitoring Living Expenses and Encouraging Healthy Nutrition Ministry of Health. 2021; Available at: https://www.gov.il/en/pages/31012021-01 Accessed 8.8.2024.

[33] FAO. Human energy requirement. Report of a Joint FAO/WHO/UNU Expert Consultation. 4. Energy requirements of children and adolescents. 2004 Available at: https://www.fao.org/4/y5686e/y5686e06.htm Accessed 9.8.2024

[34] Institute of Medicine (U.S.) Panel on Macronutrients. Dietary reference intakes for energy, carbohydrate, fiber, fat, fatty acids, cholesterol, protein, and amino acids. ; 2005. 10.1016/s0002-8223(02)90346-9 12449285

[35] Nitzan D. A call to readjust the Israeli school feeding program. IJHPR. 2023;12(1):20. 37165373 10.1186/s13584-023-00568-7 PMC10171145

[36] Central bureau of statistics. Price generator and price indices. 2021; Available at: https://www.cbs.gov.il/he/Statistics/Pages/מחוללים/מחולל-מחירים.aspx. Accessed 9.8.2024.

[37] Yan B, Anna H, William A. Global variation in the cost of a nutrient-adequate diet by population group: an observational study. The Lancet Planetary health. 2022;6(1):e19–28. 34998455 10.1016/S2542-5196(21)00285-0 PMC8753783

[38] The world bank. Food Prices for Nutrition | DataBank. 2023; Available at: https://databank.worldbank.org/source/food-prices-for-nutrition#. Accessed 9.8.2024.

[39] R Strier. Activity report of the National Food Security Council for 2023. National Council for Food Security 2023 Dec 10.

[40] Ritchie LD. School meals matter: federal policy can improve children’s nutrition and health. Public Health Nutr. 2020;23(16):3025–7. 32814605 10.1017/S1368980020002591 PMC10200607

[41] Jia J, Moore LL, Cabral H, Hanchate A, LaRochelle MR. Changes to dietary and health outcomes following implementation of the 2012 updated US Department of Agriculture school nutrition standards: analysis using National Health and Nutrition Examination Survey, 2005–2016. Public Health Nutr. 2020;23(16):3016–24. 32723401 10.1017/S1368980020001986 PMC10200493

[42] Food and Nutrition Service. National School Lunch Program (NSLP) Fact Sheet | Food and Nutrition Service. U.S. Department of Agriculture 2019. Available at: https://www.fns.usda.gov/nslp Accessed 9.8.2024

[43] Kenney EL, Barrett JL, Bleich SN, Ward ZJ, Cradock AL, Gortmaker SL. Impact Of The Healthy, Hunger-Free Kids Act On Obesity Trends. Health Aff (Millwood). 2020;39(7):1122–9. 10.1377/hlthaff.2020.00133. 32634356 10.1377/hlthaff.2020.00133 PMC7961790

[44] Darmon N, Drewnowski A. Does social class predict diet quality? Am J Clin Nutr. 2008;87(5):1107–17. 18469226 10.1093/ajcn/87.5.1107

[45] Rao M, Afshin A, Singh G, Mozaffarian D. Do healthier foods and diet patterns cost more than less healthy options? A systematic review and meta-analysis. BMJ Open. 2013;3(12):e004277. 10.1136/bmjopen-2013-004277. 24309174 10.1136/bmjopen-2013-004277 PMC3855594

[46] Gittelsohn J, Trude ACB, Kim H. Pricing strategies to encourage availability, purchase, and consumption of healthy foods and beverages: a systematic review. Prev Chronic Dis. 2017;14:E107. 29101767 10.5888/pcd14.170213 PMC5672888

[47] Peter W. Monitoring the affordability of healthy eating: a case study of 10 years of the illawarra healthy food basket. Nutrients. 2010;2(11):1132–40. 22254001 10.3390/nu2111132 PMC3257621

[48] Canada H. The contents of the 2019 national nutritious food basket. 2020; Available at: https://www.canada.ca/en/health-canada/services/food-nutrition/food-nutrition-surveillance/national-nutritious-food-basket/contents.html Accessed 9.8.2024.

[49] Ministry of Economy and industry. Comparing the cost of the basket of selected products. 2020. Available at: https://www.gov.il/he/pages/healthy-food-basket-price-comparison Accessed 9.8.2024.

[50] Leket Israel, the largest food rescue organization in Israel, releases its Sixth Annual national food waste and rescue report in israel on the topic of the urgent need for action in the treatment of food waste and rescue in Israel Ministry of Israel is NIS 3.2b Annually Protection. Available at: https://www.gov.il/en/pages/leket-israel_2020 Accessed 9.8.2024.

