# Correction: The annual feasibility and affordability of a healthy diet for families with children in Israel by income quintile and geographic area of residency

**DOI:** 10.1186/s13584-025-00687-3

**Published:** 2025-04-14

**Authors:** Naama Dgania-Yaroslaviz, Moran Blaychfeld Magnazi, Vered Kaufman-Shriqui

**Affiliations:** 1 Faculty of Health Sciences, Department of Nutrition Sciences, Ariel University, Kiryat Hamada 3, Ariel, Israel; 2 The Center for Urban Health Solutions (C-UHS), St., Michael’S Hospital, Toronto, Canada; 3 Nutrition Division, Ministry of Health, Jerusalem, Israel; 4 School of Public Health, Haifa University, Haifa, Israel

**Correction: Israel Journal of Health Policy Research (2025) 14:15**

10.1186/s13584-025-00675-7

The original publication of this article contained an error in the ‘results’ section of the abstract. The incorrect and correct information is listed in this correction article, the original article has been updated. This error only affected the abstract, the correct information was listed elsewhere in the article.

Incorrect

The average daily cost of a healthy diet for an average Israeli family was 35.5 ± 7.7 New Israeli shekels.

Correct

The average daily dietary cost per person was 35.5 ± 7.7 NIS.

